# Effect of an Online Module on Leadership on the Knowledge Acquisition of Nursing Students: A Pilot Randomized Clinical Trial Study

**DOI:** 10.1155/jonm/3769545

**Published:** 2025-09-09

**Authors:** Jucielly Ferreira da Fonseca, Maria Carolina Batista da Silva, Vinicius dos Santos Lemos Pereira, Gabriel Pedro Duarte da Silva, Fernando Hiago da Silva Duarte, Gabriela de Sousa Martins Melo de Araújo, José Adailton da Silva, Rodrigo Assis Neves Dantas, Daniele Vieira Dantas

**Affiliations:** ^1^Graduate Program in Nursing, Federal University of Rio Grande do Norte (UFRN), Natal, State of Rio Grande do Norte, Brazil; ^2^Department of Nursing, Federal University of Rio Grande do Norte (UFRN), Natal, State of Rio Grande do Norte, Brazil; ^3^Secretariat of Public Health of Rio Grande do Norte, Natal, State of Rio Grande do Norte, Brazil; ^4^Department of Public Health, Federal University of Rio Grande do Norte (UFRN), Natal, State of Rio Grande do Norte, Brazil

**Keywords:** distance education, educational technology, leadership, nursing

## Abstract

**Objective:** To evaluate the effect of an online educational module on leadership upon the knowledge acquisition of nursing students.

**Methods:** The research was conducted in two phases: methodological and experimental. In the methodological phase, specialists validated the content and appearance of the module, as well as the content of the problem situation. In the experimental phase, a pilot randomized clinical trial was conducted with 14 sixth-semester nursing students from the Federal University of Rio Grande do Norte, divided into two groups: experimental (seven students), who accessed the online module, and control (seven students), who participated in a conventional, face-to-face expository lecture within the Nursing Department.

**Results:** The results indicated significant advancements in leadership skills, particularly in the experimental group. This group demonstrated a notable increase in the appreciation of interpersonal skills, rising from 57.14% in the pretest to 85.71% in the post-test. Regarding the mediation of ethical conflicts, 85.71% of the control group and 100% of the experimental group opted for open debate in the post-test, evidencing a participatory approach. Furthermore, the experimental group showed improvement in self-identification as a leader, increasing from 42.86% to 57.14%, suggesting a positive impact on the development of leadership self-perception.

**Conclusion:** The online educational module on leadership had a positive effect on the knowledge acquisition of nursing students, evidenced by a significant increase in knowledge and skills related to leadership.

**Trial Registration:** Brazilian Clinical Trials Registry (ReBEC): RBR-2dfqmr2

## 1. Introduction

Social and cultural transformations, driven by technological advancements, have created new possibilities for interaction among people and processes. Concurrently, health systems face the challenge of delivering increasingly complex care amidst a shortage of nursing professionals. This situation aligns with global projections estimating a deficit of over ten million nurses by 2030 needed to meet worldwide health demands [1, 2].

Despite this scarcity, the World Economic Forum emphasizes that skills such as analytical thinking, problem-solving, creativity, leadership, and emotional intelligence are fundamental for contemporary professionals [3]. This underscores the need for professional training that focuses not only on technical competencies but also integrates scientific knowledge and socioemotional skills [4].

The COVID-19 pandemic demanded both technical and socioemotional skills from nurses, with leadership ability proving crucial for providing team emotional support and managing the health crisis [5, 6]. Furthermore, as the nursing field advances, market competitiveness requires professionals adept at managing both people and processes [7].

While the national curriculum guidelines for undergraduate nursing programs in Brazil emphasize competencies like healthcare provision, decision-making, communication, management, continuing education, and leadership, challenges persist in effectively implementing leadership training. These challenges often stem from outdated methodologies and inadequate faculty preparation [8].

Digital education emerges as a strategy to modernize professional training, fostering active learning through Digital Educational Technologies (DETs). Within the scope of DETs, online modules and courses are key components. They utilize Virtual Learning Environments (VLEs) to offer autonomous and inclusive learning experiences [9].

In this context, online modules and courses are essential elements of digital education. An online module represents a specific learning unit focused on designated topics, whereas an online course comprises a broader structure encompassing several interconnected modules, creating a comprehensive learning journey [9, 10].

This study utilized a previously developed online educational module on leadership, derived from a course provided for validation purposes. The significance of this work lies in its potential social impact: by helping to cultivate leadership competencies in nurses, it aims to contribute directly to the enhancement of health services. It prepares nurses to manage effectively, promote team well-being, and make sound ethical decisions when confronting real-world challenges within the hospital environment. This study aimed to evaluate the effect of an online educational module on leadership concerning the knowledge acquisition of nursing students.

## 2. Methods

This research was structured in two stages: (1) a methodological study that enabled the content and appearance validation of an educational strategy involving an online educational module and a problem situation, used as pretest and post-test instruments [11]; and (2) an experimental study, specifically a pilot single-blind, controlled Randomized Clinical Trial (RCT). This pilot RCT followed the Consolidated Standards of Reporting Trials (CONSORT) guidelines and aimed to compare the effects of an online educational module on leadership with conventional expository teaching, assessing knowledge acquisition among nursing students [12].

Pilot studies are instrumental in guiding decisions regarding the design of recruitment, measurement, and intervention strategies, proving particularly valuable in research introducing novel approaches. In this context, given the evaluation of a new educational technology and the paucity of experimental studies analyzing the impact of an online leadership module, conducting a pilot study was deemed essential before implementing a larger-scale RCT [12].

### 2.1. First Stage: Methodological Study

#### 2.1.1. Online Educational Module

The study utilized an online educational module [13] designed to enhance leadership and management competencies. This self-instructional module, with a total workload of 30 h, comprised four interactive lessons addressing crucial aspects for developing nurse leaders. The first lesson covered professional competencies, highlighting emerging skills identified in the World Economic Forum report [14], preparing students for the dynamic demands of the job market. The second lesson explored positive psychology and personal development, emphasizing the importance of shifting paradigms in the workplace, based on principles outlined in the book “The Happiness Advantage” [15]. The third lesson focused on communication, conflict mediation, leadership, and mindset change [16, 17]. The fourth and final lesson addressed conflict management and rapport-building techniques, drawing on principles described by Moore to enhance negotiation and problem-solving capabilities [18]. By integrating theoretical and practical knowledge, this educational module served as a core component of the study's intervention. Accordingly, its validation process adhered to the rigorous methodological guidelines described by Polit and Beck [19]. This process falls within the scope of methodological research, underscoring the importance of developing, validating, and verifying educational instruments in applied research settings.

#### 2.1.2. Recruitment of Specialists

The recruitment of specialists for validating the educational material followed criteria established by Benner et al. [20], which classify professionals by expertise level. These levels include novice (basic knowledge, intuitive judgment); advanced beginner (recognizes concepts quickly via practical examples); competent (uses prior knowledge and critical reasoning for precise interpretations); proficient (bases decisions on real experiences, though may face challenges in specific situations); and expert (possesses extensive experience, analytical refinement, and deep understanding of underlying theories) [21].

While the recommended number of specialists for analyzing educational materials varies in the literature, this study employed the “snowball” sampling technique. The process began with a search on the CNPq Lattes Platform using specific keywords (Leadership AND Nursing AND DETs) to identify individuals holding doctoral degrees in the field. Search filters included “database: doctors,” “nationality: Brazilian and foreign,” and “country of nationality: all.” Following initial identification, invitation letters were sent to potential candidates.

Considering Fehring's recommendations [22], defining nurse specialists as those with master's or doctoral degrees, a purposive sample of 15 experts was invited for validation. Seven agreed to participate, signing the Informed Consent Form (ICF) via Google Forms, where they also provided sociodemographic, academic, and professional details.

The validation occurred in a single round, allowing specialists 30 days to analyze the material and offer suggestions. Feedback concerning the problem situation and module structure was analyzed and incorporated, enhancing content quality and relevance.

#### 2.1.3. Construction of the Problem Situation

The problem situation employed in the study was developed based on research into nursing practice and crafted to simulate a challenging professional scenario. After specialist validation, it served as the pre- and post-test instrument to assess and compare leadership knowledge between the control group (CG) and experimental group (EG). The scenario depicted the implementation of the National Nursing Salary Floor (Piso Salarial Nacional da Enfermagem) in Brazil, detailing its financial repercussions on healthcare institutions, such as layoffs, increased workload, and a conflict-prone work environment. These changes led to more overtime, professional burnout, communication failures, and compromised patient safety. The problem situation aimed to stimulate critical reasoning for improving the work environment through effective leadership and team management strategies.

### 2.2. Second Stage: Experimental Study

This experimental phase investigated the intervention's effects by comparing the group receiving the online module (EG) with a group receiving conventional teaching (CG). Randomization was utilized for impartial group assignment, mitigating researcher bias.

#### 2.2.1. Study Setting

The study took place at the Department of Nursing, Federal University of Rio Grande do Norte (UFRN), Natal/RN Campus.

#### 2.2.2. Population and Sample

The study sample comprised 14 undergraduate nursing students in their sixth semester at the UFRN during the first academic semester of 2024. A nonprobabilistic convenience sampling strategy was utilized, consistent with the objectives of a pilot investigation. The participant number was established to evaluate the methodological viability of the proposed intervention and to generate preliminary data for subsequent, more extensive trials designed for greater statistical power and generalizability.

Participants were specifically selected based on their lack of prior formal education in leadership, as this content is formally introduced in the seventh semester through the “Health Services Management” discipline, according to the official curriculum (Pedagogical Course Project).

Eligibility criteria for inclusion were (a) being at least 18 years old; (b) current enrollment in the undergraduate nursing program at the UFRN; (c) being in the sixth semester of the program at the onset of 2024; and (d) having no previous engagement with leadership-oriented courses or activities. Conversely, the exclusion criteria were (a) possession of a prior undergraduate or postgraduate degree; and (b) previous participation in any leadership-focused courses, events, or training, so as to mitigate confounding variables arising from prior knowledge.

Participants who opted to withdraw from the study or did not fulfill all required components—including the completion of pre- and post-test instruments, workshop attendance, and full engagement with the online module—were excluded from the final analysis. This was done to safeguard the study's internal validity and maintain the integrity of the comparative data.

#### 2.2.3. Study Groups

Sixth-semester students were recruited via an offering of a 30 h extension course titled “Leadership in Nursing Course,” registered in the university's Integrated System for Academic Activities Management (SIGAA/UFRN). An invitation was circulated through the class WhatsApp group. Interested students completed an online Google Form, providing initial data and electronically signing the ICF. Following enrollment, a Google Meet video call was conducted to introduce the course and administer the pretest assessing baseline leadership knowledge. Participants completing this step were subsequently added to separate WhatsApp groups for each experimental condition, remaining unaware of their specific group assignment (blinding).

#### 2.2.4. Management of Losses and Withdrawals

Any participant losses or withdrawals during data collection were documented by the researcher. Participants who withdrew (*n* = 9) were excluded from the final analysis to maintain result integrity. Participants were informed they could withdraw at any point by stating their intention.

#### 2.2.5. Data Collection Systematization

Randomization ensured equitable participant distribution between groups and controlled for confounding variables, enhancing result precision and reliability. It was performed using www.random.org, which randomly assigned participants (numbered 1–14) to the CG and EG without researcher influence, ensuring equal allocation probability and bolstering internal validity. The resulting assignments were CG = 3, 4, 7, 9, 10, 13, 14; EG = 1, 2, 5, 6, 8, 11, 12.

#### 2.2.6. Intervention

Participants used electronic personal devices and internet connections. The pretest was administered synchronously to all participants in a Google Meet virtual room within a 20 min timeframe, without any prior provision of support materials.

Post- and pretest, participants were allocated to their randomized groups. The CG attended a face-to-face conventional lecture at the UFRN Department of Nursing. The EG accessed the online educational module. Allocations were communicated via WhatsApp and email.

The online module featured recorded lessons and a discussion forum, offering flexibility in access irrespective of location. The CG received a standard expository lecture. Both formats utilized visual aids like PowerPoint presentations.

After the intervention period, the post-test was administered to both groups face-to-face at the Department of Nursing, using Google Forms. Again, participants had 20 min and no support materials. A researcher supervised the administration to ensure consistent conditions.

#### 2.2.7. Data Collection Instrument

A Google Form was utilized as the primary data collection instrument, serving as both pretest and post-test for both groups to assess module efficacy and knowledge acquisition.

The instrument comprised two parts: (1) A registration section collecting relevant demographic, academic, and professional data. (2) The main assessment form containing the problem situation (with four associated objective questions addressing team motivation/engagement, organizational culture, conflict management, and constructive feedback) and an adapted version of the Questionnaire on Nursing Student Self-Perception in Leadership Practice (QUAPEEL) [23].

The problem situation component included six objective questions (each with four response options): ‘Faced with potential layoffs… keep the team motivated and engaged?', ‘Given signs of stress… preserves professional well-being?', ‘In an environment where conflicts… allocating scarce resources?', ‘As a leader, how would you provide constructive feedback… promoting professional development?', ‘How do you define leadership?', ‘Do you consider yourself a leader?', and ‘Select the interpersonal skills you consider necessary for a leader'.

The original QUAPEEL measures skills and attitudes for nursing leadership practice. For this study, its sociodemographic section was adapted for students. The QUAPEEL typically has three parts: sociodemographic data, leadership knowledge exploration (qualitative/quantitative), and specific leadership competencies (perceived possession vs. need for development). The third part (presumably retained in the adaptation) consists of 20 propositions assessing coaching process dimensions using a Likert-type scale: “1. Never”; “2. Rarely”; “3. Sometimes”; “4. Almost always”; “5. Always”; and “NA. Not applicable.” Scores range from 0 (lowest perception) to 100 (highest perception) of leadership practice. These combined instruments facilitated quantitative and qualitative assessment of leadership knowledge and self-perceived skills/attitudes among students. This standardized format ensured equal assessment conditions for both CG and EG.

#### 2.2.8. Pretest

The pretest application for both the CG and EG followed a structured and pedagogical format, intending to assess the participants' prior knowledge on topics such as team engagement, organizational culture, conflict management, and constructive feedback. The first part of the pretest consisted of a problem-solving situation and four objective questions, each addressing these key themes, with the purpose of stimulating reflection and critical thinking among the participants.

The use of problem-solving situations as a pedagogical strategy is highlighted for its effectiveness in enhancing students' reflective capacity, assisting them in developing essential competencies to face challenges in the work environment and nursing practice. This approach has been advocated in the literature as an important means for training critical and reflective professionals, prepared for problem-solving and the continuous improvement of nursing practice [24].

Data collection for the pretest was conducted online via Google Forms, without providing prior support material, ensuring participants responded based on their existing knowledge. The completion time was 20 min, with the researcher monitoring via Google Meet, who oversaw participation and tracked the pretest completion through the platform's chat.

This methodology aimed to ensure a consistent and transparent evaluation process, aligned with a progressive pedagogical approach that seeks to train critical nursing professionals, prepared to face the challenges of their field.

#### 2.2.9. Post-Test

Following the intervention, the same instrument used in the pretest was reapplied to evaluate the activity's impact. Unlike the remotely conducted pretest, where participants were in a virtual room, the post-test was administered in person, utilizing the digital resource, Google Forms, for its application.

Similar to the pretest, no support materials were provided beforehand. Participants were again given 20 min to complete the questionnaire. The researcher was present during the administration to ensure all participants had equal time and environmental conditions to finish the test.

Time management was rigorously controlled, and all participants successfully completed the post-test within the allocated timeframe. The comparison of pretest and post-test results allowed for a precise analysis of the intervention's effect, revealing the level of knowledge or skills acquired by participants throughout the process.

#### 2.2.10. Blinding

A single-blind design was adopted to ensure data integrity and minimize bias. In this model, participants were not aware of the experimental condition to which they were assigned, nor did the statistician responsible for the analysis have prior access to information about the profile of the CG and EG. To ensure impartiality in data processing, data were presented without any identifying markers that would distinguish group affiliation.

#### 2.2.11. Outcome Analysis

The analyzed outcomes relate to the evaluation of the effect of the online educational module regarding the acquisition of leadership knowledge among nursing students, measured by the problem-solving situation instrument and the QUAPEEL questionnaire.

### 2.3. Statistical Treatment

Data were processed using Microsoft Office Excel 2020 and SPSS 25.0 software, employing descriptive and inferential statistics. Results were presented in tables and figures. The descriptive treatment included the analysis of relative and absolute frequencies of the variables, in addition to the evaluation of the content validity index (CVI) and the Kappa test. The CVI, which must be greater than 0.80 to be considered acceptable [25], measures the acceptance of the items by the evaluators. The Kappa coefficient assesses the degree of agreement between evaluators, with values above 0.75 considered excellent, below 0.40 low, and between 0.40 and 0.75 moderate [26].

### 2.4. Legal and Ethical Aspects

The study was approved by the Research Ethics Committee of the UFRN 1 (Certificate of Ethical Appreciation no. 75851023.0.0000.5537, Opinion no. 6,599,419). Participation was voluntary, with the signing of the Free and ICF, ensuring that participants understood the objectives, risks, and benefits of the research and could withdraw at any time without prejudice. The study followed the ethical precepts of Resolution 466/2012 of the National Health Council, ensuring the protection of participants' rights and well-being. Measures were adopted to guarantee the confidentiality and anonymity of the data, with the anonymization of personal information.

## 3. Results

The results are organized into two sections: validation by expert judges and the experimental study.

### 3.1. Validation by Expert Judges

During the content validation of the online educational module, all items assessed for “objectivity” (*n* = 5) achieved an acceptable CVI, exceeding the 0.8 threshold. However, regarding the parameters of “clarity” and “relevance,” one item (20%, *n* = 1) was considered unacceptable, as its CVI value was below 0.8.

The Kappa coefficient, used to evaluate inter-rater agreement, indicated median agreement among the judges for 100% of the items related to “objectivity” (*n* = 5). For the parameters “clarity” and “relevance,” median agreement was observed for 75% of the items (*n* = 6), while excellent agreement was found for the remaining 25% of items (*n* = 2) (Table 1).

The appearance validation of the online educational module indicated that all items (*n* = 5) achieved excellent results on both the CVI and the Kappa test for the parameter “relevance.” However, the content validation of the clinical case revealed that three of the items assessed for the “relevance” parameter were deemed unacceptable (likely with CVI < 0.8). Despite this, for the parameters “clarity” and “objectivity,” all items related to the clinical case were considered acceptable regarding their CVI, although median agreement was observed via the Kappa test for these items. Even though some CVI results were acceptable, the overall median agreement suggested by the Kappa scores, particularly concerning the issues with relevance, indicated the need for another evaluation round (Table 2). Nevertheless, the limited availability of the expert judges precluded the execution of a second validation round, thereby limiting the possibility of further refinement based on additional expert feedback.

Although the relevance indices for the content validation of the problem situation fell below the ideal standard (based on CVI and Kappa scores), it is crucial to consider that significant qualitative feedback from the judges was accepted and incorporated. By providing detailed and constructive analysis, the judges suggested improvements, including modifications to the title of the problem situation itself.

Quantitative indices provide a general overview of content validity but may not fully capture the material's complexity and practical applicability. The adjustments made based on the judges' qualitative feedback significantly enhanced the material. Notably, the reformulation from “clinical case” to “problem situation,” aimed at including more practical and complex issues, addresses authentic training needs and was corroborated by positive specialist feedback. This feedback confirms that the revised content, being better aligned with professional challenges, possesses substantial educational relevance, thereby justifying its use and value despite the initial quantitative scores.

The researcher incorporated the judges' suggested adjustments based on the qualitative feedback received. Modifications directly addressed critical points, such as including questions related to care errors, prevention strategies, and the noted change from “clinical case” to “problem situation.” These adjustments aimed to improve the content's relevance and applicability, aligning it more closely with identified practical and theoretical needs. However, due to practical challenges regarding the judges' availability, conducting a second Delphi round for re-evaluation was not feasible.

In contrast, for the parameters “clarity” and “objectivity” in the problem-situation validation, 100% of the items (*n* = 4) were considered acceptable based on their CVI scores. The Kappa test indicated median agreement among judges for these parameters.

### 3.2. Experimental Study

**Figure 1:**
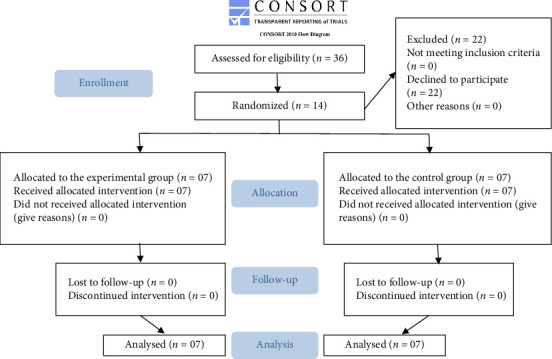
Flowchart illustrating inclusion, randomization, and analysis of research groups, based on the CONSORT 2010 guidelines.

During the data collection period, 14 participants were evaluated. They were randomized and allocated as follows: seven to the CG and seven to the EG. The participant allocation flowchart is illustrated in Figure 1.

The predominance of young adults aged 20–25 years (85.71% of participants) suggested that the group was in an early stage of their academic and professional careers. This youthfulness might be viewed positively, as these participants were likely more receptive to new leadership ideas and practices—crucial for developing skills essential in their future careers.

The finding that 92.86% of participants were female highlights a significant characteristic of the nursing field, where women represent the majority of the workforce. Lastly, the fact that all participants were enrolled in the sixth semester of their program indicates homogeneity in terms of academic progression.

Table 3 presents the data regarding the responses provided by the participants concerning the problem situation.

Regarding the approach to potential professional layoffs due to the salary floor, consistency was observed in participant responses, with 100% in both groups opting for a collaborative approach in the pretest and post-test. This choice reflects a predisposition towards maintaining open dialogue and seeking joint solutions, demonstrating a proactive and participatory stance.

When questioned about promoting an organizational culture focused on team well-being amidst signs of stress, all participants (100%) in both phases indicated a preference for customizing stress management strategies based on individual needs.

In the context of mediating conflicts arising from difficult ethical decisions, 85.71% of the CG chose open debate in both the pre- and post-test. In contrast, 100% of the EG participants selected this strategy in the post-test, suggesting a potential reinforcement of this approach by the intervention.

Regarding the provision of constructive feedback after medication errors, all participants (100%) indicated they would opt for providing individual feedback, highlighting areas for improvement and offering support. This demonstrates a uniformly positive approach towards professional development.

Concerning the definition of leadership, the data revealed a shift in the CG, with an increase from pre- to post-test in understanding leadership as a process of influence to achieve objectives. In the EG, however, the response remained stable, with 100% consistently defining leadership as “the process of exerting influence.”

Furthermore, participants' self-assessment as leaders showed a notable evolution. Self-identification as a leader increased significantly in the EG, rising from 42.86% in the pretest to 57.14% in the post-test. This suggests a positive effect of the online module on leadership self-perception and confidence.

Finally, when asked about necessary interpersonal skills for a leader, there was an increased tendency in the EG to value a combination of skills. The selection of “All the skills mentioned above” rose from 57.14% in the pretest to 85.71% in the post-test within this group. This indicates a greater awareness of essential leadership competencies and a more comprehensive recognition of the complexities involved in leading, potentially fostered by the module.

Tables 4 and 5 present the data from both the EG and CG participants regarding the QUAPEEL questionnaire responses, comparing the pretest and post-test results for the EG.

Overall, the differences between the pre- and post-test moments for the QUAPEEL items were not statistically significant, as all calculated *p* values exceeded the conventional significance level of 0.05. This implies that the observed changes between the pre- and post-test on these specific measures may be attributable to random variation rather than a statistically significant impact of the intervention.

Among the items with *p* values approaching the significance level, items 4.5 (“I contribute to effective communication in work relationships with those I lead”) and 4.6 (“I give guidance to those I lead and demonstrate how tasks should be performed”) are noteworthy, both yielding *p*=0.083. These items displayed increased means and medians in the post-test, suggesting a positive trend in the perception of these practices within the EG, although this trend did not reach statistical significance.

Additionally, some items showed notable variations in means or medians despite nonsignificant *p* values. For instance, item 4.9 (“I redirect those I lead, showing a new path forward when they do not meet expected performance”) showed an increase in the mean from 4.00 to 4.71 and the median from 4.00 to 5.00 (*p*=0.102). Another example is item 4.16 (“I make myself available to assist those I lead when they are facing professional difficulties”), where the mean increased from 4.14 to 4.83 and the median from 4.00 to 5.00 (*p*=0.180).

Table 5 presents the results for the CG. These largely indicated that the changes observed between the two periods of time (pre- and post-test) were not statistically significant, except for two items. Item 4.1 (“I know how to listen to those I lead”) demonstrated a significant improvement, with its mean increasing from 4.14 to 4.71 and the median rising from 4.00 to 5.00 (*p*=0.046). Similarly, item 4.6 (“I give guidance to those I lead and demonstrate how tasks should be performed”) also showed significant progress, with the mean increasing from 3.14 to 4.43 and the median from 3.00 to 4.00 (*p*=0.024).

Other items exhibited trends towards improvement, although they did not reach statistical significance. For item 4.5 (“I contribute to effective communication in work relationships with those I lead”), the mean increased from 4.00 to 4.43 and the median from 4.00 to 5.00 (*p*=0.083). Meanwhile, item 4.16 (“I make myself available to assist those I lead when they are facing professional difficulties”) showed growth in the mean from 4.43 to 4.83, with the median remaining stable at 5.00, but this change was not statistically significant (*p*=0.180).

Additionally, items such as 4.8 (“I recognize and value those I lead for what they do or how they behave”) and 4.19 (“I periodically monitor the results presented by each person I lead”) maintained constant means and medians between the pre- and post-test assessments (*p*=1.000), indicating no change. Conversely, item 4.20 (“I agree on the necessary deadline with each person I lead for goals to be achieved”) displayed a decrease in the mean from 4.43 to 3.83 and in the median from 5.00 to 4.00. This suggests a potential decline in this specific self-perceived practice, although the change was not statistically significant (*p*=0.083).

Overall, the results indicated significant advancements in specific areas, such as the ability to listen to those being led and offer clear guidance, while other aspects demonstrated stability or trends toward improvement.

## 4. Discussion

Analysis of the results indicated that the online leadership module was effective in enhancing nursing students' knowledge regarding a fundamental competency: leadership. Consequently, students improved their understanding of leadership concepts and their application in the contexts where they will eventually practice. This progress suggests the module contributed to consolidating knowledge that will be valuable in their future professional activities.

The CVI and Kappa analysis of the module's items revealed generally positive consistency, suggesting the course content was suitable for developing leadership competencies. Although some items showed no change or a decrease, this highlights areas that may require more in-depth focus in future educational interventions. These points towards the need for refining the educational material, aligning with findings from studies that emphasize the importance of validated materials [27, 28].

Utilizing a validation model with experts in nursing leadership ensures that the module is aligned with the training needs for developing leadership and problem-solving competencies. This approach enhances the effectiveness of the research outcomes by allowing for the progressive incorporation of feedback, which is continuously analyzed to maximize pedagogical impact, thereby making the content more useful and effective for student learning [29].

Considering current market demands, it is crucial to train and qualify nursing professionals in leadership from the beginning of their undergraduate education. Leadership is not only fundamental for professional practice but also represents an indispensable strategy for strengthening the nursing profession and expanding the roles of nurses [30, 31]. It was observed that prior to the module, only a small fraction of students self-identified as leaders. However, postintervention, self-identification increased notably in the EG. This suggests the module not only imparted technical knowledge about leadership but also fostered greater confidence among students in their capacity to fulfill this role.

The enhancement of leadership competencies is vital for the advancement of nursing, as it directly impacts the quality and effectiveness of care delivery [32]. The study suggests improvements related to attention to communication, listening, recognition, feedback, and delegation—crucial social tools for leadership. In line with this, an increased appreciation for possessing a comprehensive set of interpersonal skills was observed in the EG, with the selection of “All the skills mentioned above” rising from 57.14% in the pretest to 85.71% in the post-test.

As highlighted by Caveião et al. [33] and Amestoy et al. [34], newly graduated professionals face a significant challenge: insecurity in exercising leadership due to a lack of experience. In the pretest, 100% of the CG participants did not identify as leaders, compared to 57.14% in the EG who also did not identify as leaders. This situation changed following the interventions. In the post-test, the proportion identifying as leaders increased to 42.86% in the CG and to 57.14% in the EG. This suggests that the online module, in particular, strengthened the participants' perception of the nurse's role as a leader.

These findings indicate that the module provided more than just theoretical knowledge; it also bolstered students' confidence, enabling them to envision themselves as leaders. Therefore, implementing educational modules that encourage this self-identification is essential for training confident nurses who are prepared to meet the challenges of the contemporary job market.

The literature emphasizes the importance of innovative methods in nursing education, such as online educational modules [35–37]. The impact of the online module in fostering development not only in the understanding of leadership concepts but also in self-identification as a leader and the enhancement of other skills reinforces its relevance as a viable strategy for improving nursing competencies. Such educational practices are fundamental for advancing the training of future nurses.

This corroborates existing literature, emphasizing the need for educational strategies that engage students, promote critical thinking, and prepare future professionals for leadership roles [27, 28]. Additionally, the findings align with the goals of the Nursing Now campaign, which promotes investment in nursing leadership and encourages nurses to take on leadership positions [38]. Consequently, the study demonstrated a shift in the perception of leadership among students, particularly noticeable in the EG. They adopted a broader view, more aligned with leadership as a process of influence and mobilization aimed at achieving common goals.

This study contributed to the understanding of the effects of an online educational module on developing essential competencies, like leadership, among nursing students. The results suggest that online modules can enhance learning and students' self-identification as leaders, thereby better preparing them for the demands of the job market. This reinforces the necessity of integrating leadership training into the undergraduate curriculum, aiming to drive change, improve care delivery, and foster educational innovation that complements traditional teaching methods. The importance of nurses conducting RCTs for the growth and strengthening of nursing as a scientific discipline is also highlighted [39–41].

### 4.1. Recommendations and Implications for Practice

The findings of this study support the recommendation to integrate online, self-directed, and technology-based educational strategies into nursing curricula. This approach is particularly relevant for the cultivation of leadership competencies, which are essential for graduate nurses entering the professional workforce. As evidenced by the greater knowledge acquisition among participants in the digital intervention group, the use of online modules can enhance student engagement and facilitate the meaningful learning of management-related content.

Such digital tools represent not only viable and effective alternatives for remote education but also serve as valuable supplements to face-to-face instruction. Their integration can contribute to graduating professionals who are better equipped to navigate the complex organizational and interpersonal challenges inherent in the healthcare environment. This study's results underscore the imperative to introduce leadership concepts earlier in the nursing program and to weave them transversally throughout the curriculum instead of limiting them to specific management subjects.

### 4.2. Current Challenges and Limitations

The study is subject to certain limitations. The small sample size and the single-institution design restrict the external validity and generalizability of the results. Additionally, logistical constraints, specifically scheduling conflicts among the expert judges, precluded a second validation round, thereby limiting opportunities to refine the educational module based on a formal consensus. Nevertheless, the qualitative feedback from the experts, particularly regarding the problem-based scenario, proved invaluable for enhancing the module's content and practical applicability.

Furthermore, the self-directed format of the intervention demanded a significant degree of student autonomy and commitment. In some instances, this necessitated proactive follow-up and reminders from the research team to ensure completion. These operational hurdles are indicative of the broader challenges associated with the implementation of educational technologies within academic contexts.

## 5. Conclusion

Based on the conducted study, it was confirmed that the online educational module on leadership positively impacted the training of nursing students. The primary findings demonstrating the online module's effect include the strengthening of participants' leadership knowledge, particularly evidenced by: an increase in self-perception as a leader; enhanced appreciation of interpersonal skills; improvement in constructive feedback practices and individualized support following errors; and advancement in understanding leadership as a process of influence.

## Figures and Tables

**Table 1:** Content validity index (CVI) and kappa test for the content and appearance instrument of the online educational module (*n* = 7 judges).

Content validation of the online educational module	CVI	Kappa
*1. Objectivity*		
a. The learning objectives of the online educational module are aligned with what was proposed.	0.71	0.52
b. The concepts are appropriate for the target audience.	0.71	0.52
c. The didactic structure of the course contributes to learning.	0.86	0.71
d. The support material was relevant for clarifying doubts, including after course completion.	0.71	0.52
e. The media resources (figures/images, links, animations, quizzes) facilitate learning about the topic.	0.86	0.71
*2. Clarity*		
a. Navigation instructions (page-to-page, section-to-section, link-to-link) are easy to understand.	0.86	0.71
b. Course content contributes to nursing students'/professionals' practice in leadership contexts.	0.57	0.43
c. The didactic and content structure of the course is adequate.	0.71	0.52
d. Pedagogical resources are consistent with the proposed objectives.	0.86	0.71
e. The execution speed of media resources is adequate.	1.00	1.00
*3. Relevance*		
a. Pages are suitable for the types of information presented.	0.86	0.71
b. The course proposes different learning situations.	0.57	0.43
c. I would like to continue using the course material to study the topic.	0.71	0.52

**Appearance validation of the online educational module**	**CVI**	**Kappa**

*1. Relevance*		
a. The identity/layout of the course is presented attractively.	1.00	1.00
b. The online educational module is easily accessible.	1.00	1.00
c. The virtual environment features a responsive design, adapting well to any access device.	1.00	1.00
d. The audio quality of the lessons is good.	1.00	1.00
e. Access to media resources (figures/images, links, animations, games) is fast.	1.00	1.00

**Table 2:** Content validity index (CVI) and kappa test for the content and appearance instrument of the problem situation (*n* = 7 judges).

Content validation of the problem situation	CVI	Kappa
*1. Relevance*		
a. The clinical case fulfills the purpose of simulating a scenario where the student must apply leadership knowledge.	0.57	0.43
b. The clinical case is pertinent regarding the concept of leadership.	0.57	0.43
c. The clinical case provides learning through a problem-based approach.	0.57	0.43

*1. Clarity*		
a. The description of events in the clinical case is presented in an organized and logical manner.	0.71	0.52
b. The language used is easy to understand.	0.86	0.71

*1. Objectivity*		
a. The facts in the clinical case were presented neutrally, without personal opinions or judgments.	0.86	0.71
b. The language used describes the facts objectively.	0.86	0.71

**Table 3:** Number of respondents according to information related to the course “Developing skills for leadership.”

Variables	Control group				Experimental group			
	Pretest		Post-test		Pretest		Post-test	
	*n*	%	*n*	%	*n*	%	*n*	%
*1. Faced with potential layoffs of nursing professionals due to the salary floor, how would you, as a leader, address this issue to keep the team motivated and engaged?*								
	*n*	%	*n*	%	*n*	%	*n*	%
Ignore the situation, focusing only on clinical aspects.	0	0.0	0	0.0	00	0.0	0	0.0
Seek open dialogue, exploring collaborative solutions.	7	100.00	7	100.0	7	100.0	7	100.0
Adopt an authoritarian stance to avoid salary discussions.	0	0.0	0	0.0	00	0.0	0	0.0
Delegate problem resolution to administration, without getting involved.	0	0.0	0	0.0	00	0.0	0	0.0

*2. Given signs of stress in the team, how would you promote an organizational culture that effectively addresses workload and preserves professional well-being?*								
	*n*	%	*n*	%	*n*	%	*n*	%
Implement a single stress management program for the entire team.	0	0.0	0	0.0	00	0.0	0	0.00
Ignore the stress, focusing only on clinical goals.	0	0.0	0	0.0	00	0.0	0	0.0
Increase the workload to accelerate the adaptation process.	0	0.0	0	0.0	0	0.0	0	0.0
Customize stress management strategies based on individual team members' needs.	7	100.00	7	100.00	7	100.00	7	100.00

*3. In an environment where conflicts may arise from difficult ethical decisions, how would you, as a leader, mediate a conflict between team members with opposing views on allocating scarce resources?*								
	*n*	%	*n*	%	*n*	%	*n*	%
Avoid taking sides, letting the team resolve the conflict alone.	0	0.0	0	0.0	0	0.0	0	0.0
Promote open debate and encourage the joint search for ethical solutions.	6	85.71	6	85.71	7	100.00	7	100.00
Make a unilateral decision to avoid prolonging the conflict.	1	14.29	1	14.29	0	0.0		0.0
Ignore the conflict, focusing only on clinical issues.	0	0.0	0	0.0	0	0.0	0	0.0

*4. As a leader, how would you provide constructive feedback to team members who made medication errors, while simultaneously promoting professional development?*								
	*n*	%	*n*	%	*n*	%	*n*	%
Ignore the errors to avoid internal conflicts.	0	0.0	0	0.0	0	0.0	0	0.0
Provide feedback in an individual meeting, highlighting areas for improvement and offering support for development.	7	100.0	7	100.0	7	100.0	7	100.0
Publicly hold the involved professionals accountable, aiming to prevent future errors.	0	0.0	0	0.0	0	0.0	0	00
Exclude the involved professionals, considering the errors unacceptable.	0	0.0	0	0.0	0	0.0	0	00

*5. How do you define leadership?*								
	*n*	%	*n*	%	*n*	%	*n*	%
The process of influencing people's behavior to achieve objectives in specific situations.	6	85.71	7	100	7	100.00	7	100.00
The process of transforming the behavior of an individual or an organization.	0	0.00	0	0.00	0	0.00	0	0.00
The legitimate right to exercise power within the organization to obtain workers' obedience.	0	0.00	0	0.00	0	0.00	0	0.00
I think it would be more of a mix between the first and second options.	1	14.29	0	0.00	0	0.00	0	0.00

*6. Do you consider yourself a leader?*								
	*n*	%	*n*	%	*n*	%	*n*	%
Yes	0	0.00	3	42.86	3	42.86	4	57.14
No	7	100.0	4	57.14	4	57.14	3	42.86

*7. Select the interpersonal skills you consider necessary for a leader?*								
	*n*	%	*n*	%	*n*	%	*n*	%
Communication skills.	2	28.58	2	28.58	3	42.86	1	14.29
Skill in giving and receiving feedback.	0	0	0	0	0	0	0	0
Skill in gaining power and exerting influence.	0	0	0	0	0	0	0	0
All the skills mentioned above.	5	71.45	4	71.45	4	57.14	6	85.71

**Table 4:** Comparison of item means for “Developing Skills for Leadership” (QUAPEEL items) in the pre- and post-test, using the Wilcoxon signed-rank test for paired samples for the experimental group.

Items	Pretest			Post-test			*p*
Leadership practice	Mean	Median	Std. Dev.	Mean	Median	Std. Dev.	
4.1. I know how to listen to those I lead.	4.71	5.00	0.49	4.57	5.00	0.54	0.317
4.2. I manage to maintain the interest of those I lead in maintaining and continuing dialogue.	3.43	4.00	0.79	4.00	4.00	0.89	0.102
4.3. I transmit guidance and advice to those I lead, addressing their professional needs.	4.00	4.00	1.00	4.17	4.00	0.75	1.000
4.4. I use verbal communication and pay attention to nonverbal communication in dialogue with those I lead.	4.29	5.00	0.95	4.57	5.00	0.54	0.317
4.5. I contribute to effective communication in work relationships with those I lead.	4.00	4.00	0.58	4.50	4.50	0.55	0.083
4.6. I give guidance to those I lead and demonstrate how tasks should be performed, according to their needs.	3.86	4.00	0.90	4.29	4.00	0.49	0.083
4.7. I clarify doubts of those I lead regarding their tasks.	4.14	4.00	0.90	4.71	5.00	0.49	0.102
4.8. I recognize and value those I lead for what they do or how they behave.	4.57	5.00	0.79	4.43	4.00	0.54	0.705
4.9. I redirect those I lead, showing a new path forward when they do not meet expected performance.	4.00	4.00	0.82	4.71	5.00	0.49	0.102
4.10. I periodically monitor the performance of those I lead.	4.25	4.50	0.96	4.17	4.00	0.75	0.655
4.11. I encourage the practice of feedback with those I lead.	4.50	5.00	0.84	4.33	4.00	0.52	0.564
4.12. I exert influence on those I lead, expanding competencies in favor of effective results.	4.00	4.00	0.89	4.57	5.00	0.54	0.102
4.13. I share decisions with those I lead.	4.43	4.00	0.54	4.83	5.00	0.41	0.157
4.14. I delegate activities to those I lead, sharing responsibilities.	4.17	4.00	0.75	4.50	5.00	0.84	0.257
4.15. I take responsibility for the development of those I lead.	4.50	4.50	0.55	4.50	4.50	0.55	1.000
4.16. I make myself available to assist those I lead when they are facing professional difficulties.	4.14	4.00	0.90	4.83	5.00	0.41	0.180
4.17. I ask for the opinion of those I lead to alter a procedure or propose an operational change.	4.00	4.00	0.82	4.33	4.00	0.52	0.655
4.18. I assist in defining goals for each person I lead on my team.	4.14	4.00	0.69	4.67	5.00	0.52	0.257
4.19. I periodically monitor the results presented by each person I lead.	4.00	4.00	0.89	4.40	5.00	1.34	0.564
4.20. I agree on the necessary deadline with each person I lead for goals to be achieved.	4.20	5.00	1.09	4.17	4.50	1.17	0.655

**Table 5:** Comparison of item means for “Developing Skills for Leadership” (QUAPEEL items) in the pre- and post-test, using the Wilcoxon signed-rank test for paired samples for the control group.

Items	Pretest			Post-test			*p*
Leadership practice	Mean	Median	Std. Dev.	Mean	Median	Std. Dev.	
4.1. I know how to listen to those I lead.	4.14	4.00	0.69	4.71	5.00	0.49	0.046
4.2. I manage to maintain the interest of those I lead in maintaining and continuing dialogue.	3.71	4.00	0.95	3.86	4.00	0.38	0.564
4.3. I transmit guidance and advice to those I lead, addressing their professional needs.	3.43	3.00	0.98	4.00	4.00	0.58	0.194
4.4. I use verbal communication and pay attention to nonverbal communication in dialogue with those I lead.	4.29	4.00	0.49	4.14	4.00	0.69	0.564
4.5. I contribute to effective communication in work relationships with those I lead.	4.00	4.00	0.58	4.43	5.00	0.79	0.083
4.6. I give guidance to those I lead and demonstrate how tasks should be performed, according to their needs.	3.14	3.00	0.69	4.43	4.00	0.54	0.024
4.7. I clarify doubts of those I lead regarding their tasks.	4.14	4.00	0.90	4.43	4.00	0.54	0.157
4.8. I recognize and value those I lead for what they do or how they behave.	4.57	5.00	0.54	4.57	5.00	0.54	1.000
4.9. I redirect those I lead, showing a new path forward when they do not meet expected performance.	3.71	4.00	0.49	4.00	4.00	0.58	0.317
4.10. I periodically monitor the performance of those I lead.	3.86	4.00	0.38	4.00	4.00	0.82	0.564
4.11. I encourage the practice of feedback with those I lead.	4.43	5.00	0.79	4.29	4.00	0.76	0.785
4.12. I exert influence on those I lead, expanding competencies in favor of effective results.	4.14	4.00	0.69	4.00	4.00	0.82	0.705
4.13. I share decisions with those I lead.	4.29	4.00	0.49	4.14	4.00	0.69	0.564
4.14. I delegate activities to those I lead, sharing responsibilities.	4.14	4.00	0.90	4.29	4.00	0.49	0.705
4.15. I take responsibility for the development of those I lead.	3.86	4.00	0.69	4.14	4.00	0.69	0.480
4.16. I make myself available to assist those I lead when they are facing professional difficulties.	4.43	5.00	0.79	4.83	5.00	0.41	0.180
4.17. I ask for the opinion of those I lead to alter a procedure or propose an operational change.	4.43	5.00	0.79	4.43	4.00	0.54	1.000
4.18. I assist in defining goals for each person I lead on my team.	4.00	4.00	0.58	4.14	4.00	0.69	0.317
4.19. I periodically monitor the results presented by each person I lead.	4.00	4.00	1.00	4.00	4.00	0.82	1.000
4.20. I agree on the necessary deadline with each person I lead for goals to be achieved.	4.43	5.00	0.79	3.83	4.00	0.41	0.083

## Data Availability

The data that support the findings of this study are openly available in UFRN Institutional Repository at https://repositorio.ufrn.br/jspui/handle/123456789/62505, 2024.

## Supporting Information

Supporting Information 1 S1: Ethics Committee Opinion.

Supporting Information 2 S2: ReBEC.

Supporting Information 3 S3: Statistical Report.

Supporting Information 4 S4: Checklist Consort.

Supporting Information 5 S5: CONSORT Flow diagram.

Supporting Information 6 S6: Certificate English Editing.
