# Supplementary material for: Effect of an Online Module on Leadership on the Knowledge Acquisition of Nursing Students: A Pilot Randomized Clinical Trial Study
Source: J Nurs Manag. 2025 Sep 9;2025:3769545. doi: 10.1155/jonm/3769545 (PMC12440644; doi:10.1155/jonm/3769545)
Supplement: Supporting Information 1 — S1: Ethics Committee Opinion. [file 3769545.f1.pdf]

Machine Translated by Google

CONSUBSTANTIATED OPINION OF THE CEP

RESEARCH PROJECT DATA

Search Title: EFFECT OF ONLINE MODULE ON KNOWLEDGE ACQUISITION NURSING STUDENTS ON LEADERSHIP: clinical trial

Researcher: DANIELE VIEIRA DANTAS

Thematic Area:

Version: 2

CAAE: 75851023.0.0000.5537

Proposing Institution: Nursing Department

Main Sponsor: Own Financing

OPINION DATA

Opinion Number: 6,599,419

Project Presentation:

This is a Master's-level research protocol proposed by the Postgraduate Program in Nursing. The research originates from a master's project. It will be carried out in the Nursing Department at the Federal University of Rio Grande do Norte - Natal Campus. The target audience will be seventh-semester Nursing students. The research will be a two-phase study: in the first phase, a methodological study will be carried out, which will enable the validation of the content and appearance of the online educational module, as well as, will allow the validation of the content of the clinical case carried out by judges. In the second phase, an experimental study will be carried out, of the Randomized Clinical Trial type, in which the students will be randomly distributed, into an experimental group, which will receive the intervention through digital educational technology, and a control group, which will receive a standard intervention consisting of an expository class. In these scenarios, a pre-test and post-test instrument will be applied, which corresponds to a clinical case. In addition to providing an expansion of studies and knowledge about leadership in the area of Nursing, this research will enable actions/ interventions to be developed based on the results obtained that promote more effective learning about leadership in the training context, providing students with greater knowledge and increased confidence in the subject, forming a nurse capable of promoting an efficient and collaborative work environment, optimizing results.

Machine Translated by Google

achieved in patient care and contributing to the success of the healthcare institution as a whole.

In addition to validating instruments that become valuable assets for the research community, they can be shared and used in other studies, saving time and resources for other researchers. Sixty individuals will participate in the research and will be divided into three groups: 27 in the control group, 27 in the intervention group and six as judges.

Research Objective:

Hypothesis: Null hypothesis (HO): the use of an online educational module is not effective for the acquisition of knowledge of Nursing students about Leadership. Alternative hypothesis (H1): the use of an online educational module is effective for the acquisition of knowledge of Nursing students about Leadership.

Primary Objective:

To evaluate the effectiveness of an online educational module in the acquisition of knowledge by students of Nursing about Leadership.

Risk and Benefit Assessment:

Risks:

One risk is that the results of the research may not be aligned with participants' expectations. If students do not perceive improvements in their leadership skills, this could lead to feelings of frustration or demotivation, given that they have invested their time and effort in the research. In addition, the assessment process itself may raise concerns, such as embarrassment due to the exposure of their leadership skills, and in some cases, it is recognized that participation in the research may require additional time and effort from students, especially in the experimental group, due to the follow-up of the educational module. Therefore, measures will be taken to minimize the negative impact, including flexibility in schedules and a conducive environment that allows participants to feel comfortable participating. Ongoing communication with participants will also be maintained so that they can express concerns and receive support as needed, thus ensuring safe and rewarding participation in the research. For those who feel

Machine Translated by Google

Those who are harmed by the research will be offered extra tutoring, conducted by the researcher, to ensure the opportunity to learn meaningfully about leadership concepts, regardless of the outcome of the research. This is to ensure that the experience of participating in the research is educational and enriching, regardless of the specific results. It is important to note that at the end of the research, the control group will receive the same treatment as the experimental group.

Benefits:

It should be noted that this research will provide direct benefits to participants, as it will expand the knowledge about leadership. Thus, based on the results obtained, actions/interventions can be developed that promote more effective learning about leadership in the training context, providing the student with greater knowledge and increased confidence in the subject, forming a nurse capable of promoting an efficient and collaborative work environment, optimizing the results achieved in patient care and contributing to the success of the health institution as a whole.

Furthermore, it is important to emphasize that this research is not limited to the direct benefits it provides. It also indirectly contributes to the advancement of the academic and scientific community as a whole. During the development of the research, a fundamental part of the work is the creation of assessment instruments, which will be rigorously developed and validated. This validation ensures that such instruments are reliable and accurate in measuring the variables under study. Once validated,

These instruments become valuable assets for the research community, and can be shared and used in other studies, saving time and resources for other researchers. This process of content and appearance evaluation ensures that the instruments meet the highest quality standards, increasing their usefulness and credibility in future investigations. Therefore, the benefits of this research extend beyond the immediate scope of the project, contributing to the advancement of knowledge

in your field of study.

It is crucial to emphasize that the research is conducted with the utmost ethics and responsibility towards the participants. With regard to the students who make up the control group, the researcher undertakes to ensure that, after the conclusion of the research, they will also have the opportunity to access
the online educational module.

This initiative reflects the concern for the well-being of participants, ensuring that all students involved in the research have equal opportunities in terms of learning.

Machine Translated by Google

and development. By providing access to the educational module to control group participants, the researcher demonstrates an ongoing commitment to the academic and personal growth of students, regardless of their assignment to the experimental or control group.

This approach not only balances the ethical treatment of participants, but also promotes a fair and inclusive research environment where everyone has the chance to benefit from the information and educational material provided. This not only respects the rights of participants, but also contributes to the enrichment of the research process by ensuring that all students have the opportunity to acquire knowledge relevant to their academic and professional development.

Comments and Considerations on the Research:

The research is interesting and focuses on the need to evaluate the impact of an online educational module aimed at Nursing students, focusing on the theme of leadership.

Considerations on Mandatory Presentation Terms: In the current version of the research protocol, the mandatory presentation terms comply with current ethical regulations and resolutions.

Recommendations:

The researcher must submit partial and final research reports. See models at <www.cep.propesq.ufrn.br>.

Any change to the approved protocol must first be requested through an amendment via the Brazil Platform. See manuals at <www.cep.propesq.ufrn.br>.

Conclusions or Pending Issues and List of Inadequacies:

The researcher in charge complied with the requests made in the Consolidated Opinion number 6,539,349, which included the following pending issues:

1) In the risks field of the PB research project (project generated on the Brasil platform) explain the ways to minimize risks.

Machine Translated by Google

2) In the TCLE, number the pages. Ex.: if the document has three pages 1/3,2/3,3/3.

3) Make it clear, in the TCLE, that at the end of the research, the control group will receive the same treatment as the experimental group, if this is effective.

Considering that all the aforementioned issues have been resolved, the Research Ethics Committee - CEP, in accordance with the attributions defined in CNS Resolution No. 466 of 2012, and with Operational Standard No. 001, of 2013, of the CNS, is in favor of approving the research protocol in question.

Final Considerations at the discretion of the CEP:

In accordance with Resolution 466/12 of the National Health Council - CNS and the Manual Operational for Ethics Committees - CONEP is the responsibility of the responsible researcher:

1. Prepare the Free and Informed Consent Form — TCLE — in two copies, initialed on all sides. its pages and signed, at the end, by the person invited to participate in the research, or by his/her legal representative, as well as by the responsible researcher, or by the person(s) delegated by him/her, with the signature pages being on the same sheet (Res. 466/12 - CNS, item IV.5d);

2. Develop the project as outlined (Res. 466/12 - CNS, item XI.2c);

3. Submit any amendments or extensions to the CEP with justification (Operational Manual for Ethics Committees - CONEP, Brasília - 2007, p. 41);

4. Discontinue the study only after analysis and manifestation, by the CEP/CONEP/CNS/MS System that approved it, of the reasons for such discontinuation, except in cases of justified urgency for the benefit of its participants (Res. 446/12 - CNS, item III.2u);

5. Prepare and present partial and final reports (Res. 446/12 - CNS, item XI.2d);

Machine Translated by Google

6. Keep the research data in a physical or digital file, under your care and responsibility, for a period of 5 years after the end of the research (Res. 446/12 - CNS, item XI.2f);

7. Forward the research results for publication, with due credit to the associated researchers and technical staff involved in the project (Res. 446/12 - CNS, item XI.2g) and,

8. Justify, with reasoned justification, before the CEP or CONEP, the interruption of the project or non-publication of the results (Res. 446/12 – CNS, item XI.2h).

This opinion was prepared based on the documents listed below:

Type Document File PB_INFORMAÇÕES_BÁSICAS_DO_P Basic		Post	Author	Situation
Carta_de_Respostas_as_Pendencias.pd f	Information of the Project ROJETO_2240032.pdf Others	05/12/2023 11:20:18		Accepted
PB_INFORMAÇÕES_BÁSICAS_DO_P Basic Information of the Project ROJETO_2240032.pdf		05/12/2023 11:19:02	DANIELE VIEIRA DANTAS	Accepted
Justification of Absence TCLE / Terms of Consent / Justification of	TCLE / Terms of TCLE_Juizes_modificado.docx Consent / Absence Detailed Project / Brochure	05/12/2023 10:57:01		Accepted
Investigator Others		05/12/2023 10:46:59	DANIELE VIEIRA DANTAS	Accepted
	TCLE_participants_modified.docx	05/12/2023 10:43:17	DANIELE VIEIRA DANTAS	Accepted
		07/11/2023 00:07:46	DANIELE VIEIRA DANTAS	Accepted
	EFFECT_OF_LEADERSHIP_MODULE_ON_THE_ACQUISITION_OF_KNOWLEDGE_IN_NURSING_STUDIES.pdf Co[illegible]	06/11/2023 23:28:41	DANIELE VIEIRA DANTAS	Accepted
Others	Researcher_identification_sheet .pdf	06/11/2023 23:27:53	DANIELE VIEIRA DANTAS	Accepted
Others	letter_of_consent.pdf	06/11/2023 23:25:08	DANIELE VIEIRA DANTAS	Accepted
Others	Declaration_of_Ethical_Commitment_of_Non-Start.pdf	03/11/2023 10:01:03	DANIELE VIEIRA DANTAS	Accepted
Timeline	research_schedule.pdf	03/11/2023 09:52:47	DANIELE VIEIRA DANTAS	Accepted

Address: Federal University of Rio Grande do Norte (UFRN). Sen. Salgado Filho Ave., 3000.
Neighborhood: Lagoa Nova **Zip Code: Municipality:** NATAL 59.078-900
State: RN
Telephone: (84)99193-6266 **Email:** cepufrn@reitoria.ufrn.br

Machine Translated by Google

Title Page	Cover page_Jucielly_Fonseca_signed_.pdf	02/11/2023 18:56:42	DANIELE VIEIRA DANTAS	Accepted

Opinion Status:
Approved

Needs CONEP's Appreciation:
No

CHRISTMAS, December 22, 2023

Signed by:
PAULA FERNANDA BRANDÃO BAPTIST OF SANTOS
(Coordinator)
