# Supplementary material for: Effect of an Online Module on Leadership on the Knowledge Acquisition of Nursing Students: A Pilot Randomized Clinical Trial Study
Source: J Nurs Manag. 2025 Sep 9;2025:3769545. doi: 10.1155/jonm/3769545 (PMC12440644; doi:10.1155/jonm/3769545)
Supplement: Supporting Information 3 — S3: Statistical Report. [file 3769545.f3.docx]

1. **Methodology**

The database was built in EXCEL format, version 2020. To create descriptive tables and apply statistical tests, the statistical software SPSS, version 25.0, was used.

1. **Statistical methods**

To evaluate the Content and Appearance Validation Scale, as well as the problem situation of "Developing Leadership Skills", the absolute frequency and percentage were calculated. In addition, in the "Exercised Leadership Practice" module, a descriptive statistical analysis was performed, considering the mean, median and standard deviation of the data obtained in the pre- and post-test.

To evaluate the Content and Appearance Validation instrument, the objectivity, clarity and relevance modules were applied to calculate the Content Validation Index (CVI) in order to determine the percentage of agreement among experts. Additionally, the Kappa test was used to measure the degree of agreement among experts in these same modules.

Regarding the problem situation, the McNemar test was applied to compare the pre- and post-test results in the Control and Experimental Groups. To compare the scores of the intervention and control groups in the chapter "Exercised Leadership Practice", the Wilcoxon test for paired samples was applied. This test was chosen due to the violation of the assumptions required by parametric tests, such as normality, homoscedasticity (constant variance) and independence of data. The results were presented in tables for better visualization and interpretation. All statistical tests were performed considering a significance level of 5%.

**2.1 Content Validity Index (CVI)**

It is a measure used to assess the validity of a research instrument, considering its suitability for the proposed objectives. This index is calculated based on the evaluation of experts in the field of study, who analyze the items of the instrument and provide their opinions on the relevance and clarity of each one. The CVI is determined by the percentage of experts who agree on the validity of the items. The higher the CVI value, the greater the agreement among experts regarding the validity of the instrument's content. This index is essential to ensure that the instrument is appropriate and reliable for the context in which it will be applied. The following equation presents the calculation of the CVI .

$CVI =\frac{nunber of adequate responses}{total number of responses}$

**2.2 Kappa test**

The Kappa test (or Kappa index) is a statistical measure used to assess the degree of agreement or consistency between two or more raters who are independently rating or categorizing items. This test is useful when you want to measure agreement in situations where the categories are qualitative, such as when evaluating responses in a questionnaire.

The interpretation of Kappa values ​​can be categorized as follows: values ​​below 0 indicate no or negative agreement, between 0 and 0.20 indicate weak agreement, from 0.21 to 0.40 moderate agreement, from 0.41 to 0.60 substantial agreement, from 0.61 to 0.80 strong agreement, and values ​​between 0.81 and 1 indicate almost perfect agreement, according to Cohen (1960). This index is essential to ensure the consistency and reliability of the classifications made by the evaluators, helping to ensure the quality of the data in studies and research. The formula for the Kappa index is as follows:

$k=\frac{P_{0}-P_{e}}{1-P_{e}}$

Being:

**P_o_:** (observed agreement) is the proportion of agreements observed between the evaluators. It is calculated by adding the number of classifications in which the evaluators agreed and dividing by the total number of classifications.

**P_e_** (expected agreement) is the proportion of agreements that would be expected by chance. Calculated based on each rater's marginal distributions.

The observed agreement P_o_​ is given by: $P_{0}=\frac{\sum_{i} n_{i}}{N}$

Being:

n_i_: number of ratings on which the raters agreed for category i,

N: total number of ratings.

The expected agreement Pe is given by: $P_{e}=\sum_{i} \left( \frac{(A_{i}xB_{i})}{N^{2}} \right)$

Being:

A_i_ is the proportion of ratings for category i by the evaluator 1,

B_i_ is the proportion of ratings for category i by the evaluator 2,

N is the total number of ratings.

**2.3 McNemar test**

The McNemar test is a statistical test used to assess changes in paired data, that is, to verify whether there is a significant difference between two categorical variables at two different times, usually in the context of before and after data, or of an intervention and control group. This test is particularly indicated for dichotomous data (allowing the assessment of symmetry or changes in the responses of the same sample. (Siegel; Castellan, 1988).

Test hypothesis

Null Hypothesis (H₀): There is no significant difference between the proportions of disagreements in the pre and post test.

Alternative Hypothesis (H1): There is a significant difference between the proportions of disagreements in the pre- and post-test.

$x^{2}=\frac{(b-c)^{2}}{b+c}$

Being:

b is the number of disagreements (individuals who changed from Category 1 to Category 2)

c is the number of disagreements (individuals who changed from Category 2 to Category 1)

**2.4 Wilcoxon test**

The Wilcoxon test for paired samples or dependent is a nonparametric statistical test used to compare two related measurements, for example, before and after a treatment. The test is a nonparametric alternative to the paired samples t-test and is useful when the data do not follow a normal distribution. The objective is to verify whether there is a significant difference between pairs of observations. The Wilcoxon test evaluates the sum of the ranks of the absolute differences between pairs, considering whether these differences are positive or negative (Siegel; Castellan, 1988).

Wilcoxon Test Hypotheses

Null Hypothesis (H₀): There is no difference in the before and after measurements (or in the two conditions compared). That is, the median of the differences is zero.

Alternative Hypothesis (H₁): There is a significant difference in the before and after measurements (or in the two conditions compared). That is, the median of the differences is not zero.

1. **Results**

| **Table 1: Validation of the online educational module** | | | | | |
| --- | --- | --- | --- | --- | --- |
| **Content validation** | |  | |  |  |
| **1. Objectivity** | **n** | | **CVI_1_** | | ***Kappa*** |
| a. The online educational module has learning objectives aligned with what was proposed. | | 05 | | 0,71 | 0,52 |
| b.The concepts covered are appropriate for the target audience | | 05 | | 0,71 | 0,52 |
| c. The course's didactic structure contributes to learning the content. | | 06 | | 0,86 | 0,71 |
| d. The support material was relevant to clarify doubts, even after completing the course. | | 05 | | 0,71 | 0,52 |
| e. Media resources (figures/images, links, animations, quizzes) facilitate learning about the topic in question. | | 06 | | 0,86 | 0,71 |
| **2. Clarity** | |  | |  |  |
| a. It's easy to understand navigation guidelines, page by page, section by section, or from one link to another, without getting lost or confused. | | 06 | | 0,86 | 0,71 |
| b. The content presented in the course contributes to the performance of Nursing students/professionals in the context of leadership. | | 04 | | 0,57 | 0,43 |
| c. The course's didactic and content structure is adequate. | | 05 | | 0,71 | 0,52 |
| d. The pedagogical resources are in line with the proposed objectives. | | 06 | | 0,86 | 0,71 |
| e. The speed of execution of media resources is adequate. | | 07 | | 1,00 | 1,00 |
| **3. Relevance** | |  | |  |  |
| a. The pages are suitable for the types of information presented. | | 06 | | 0,86 | 0,71 |
| b. The course proposes different learning situations. | | 04 | | 0,57 | 0,43 |
| c. I would like to continue using the course material to study the topic. | | 06 | | 0,71 | 0,52 |
| **Appearance validation** | | | | | |
| 1. **Relevance** | | **n** | | **CIV** | ***Kappa*** |
| a. The course identity/layout is presented in an attractive way. | | 07 | | 1,00 | 1,00 |
| b. The online educational module is easily accessible. | | 07 | | 1,00 | 1,00 |
| c. The virtual environment features a responsive design, adapting well to any access device (cell phone, tablet, computer). | | 07 | | 1,00 | 1,00 |
| d. The audio of the classes is welcome. | | 07 | | 1,00 | 1,00 |
| e. Access to media resources (figures/images, links, animations, games) is quick. | | 07 | | 1,00 | 1,00 |
| **Validation of the problem situation** | | | | | |
| **Content validation** | |  | |  |  |
| - - - 1. **Relevance** | | **n** | | **CIV** | ***Kappa*** |
| a. The clinical case serves the purpose of simulating a scenario in which the student has to use knowledge on the topic of leadership. | | 04 | | 0,57 | 0,43 |
| b.The clinical case is pertinent to the concept of leadership. | | 04 | | 0,57 | 0,43 |
| c. The clinical case provides learning through problem. | | 04 | | 0,57 | 0,43 |
| 1. **Clarity** | |  | |  |  |
| a. The description of events in the clinical case are described in an organized and logical manner. | | 05 | | 0,71 | 0,52 |
| b. The language used is easy to understand. | | 06 | | 0,86 | 0,71 |
| **3. Objectivity** | |  | |  |  |
| a.The clinical case had the facts presented in a neutral way, without adding personal opinions or judgments. | | 06 | | 0,86 | 0,71 |
| b. The language used is describing the facts. | | 06 | | 0,86 | 0,71 |

Source: prepared by the author himself (1) Content Validity Index

The validation of the content of the online educational module showed that all items (n=5) achieved an acceptable content validity index in the “objectivity” parameter. However, with regard to “clarity” and “relevance”, one item (20%, n=1) was considered unacceptable, since the value was below 0.8. The Kappa test, which aims to assess the agreement between evaluators, indicated that, for the “objectivity” parameter, there was median agreement in 100% of the items (n=5). For the “clarity” and “relevance” parameters, it was observed that 75% of the items (n=6) showed median agreement, while 25% of the items (n=2) showed excellent agreement.

For the appearance validation of the online educational module, it was shown that all items (n=5) achieved an excellent Content Validity Index and Kappa test for the “relevance” parameter. However, validation of the clinical case content revealed that three of the items evaluated for the "relevance" parameter were considered unacceptable. Despite this, for the "clarity" and "objectivity" parameters, all items were considered acceptable, with median agreement observed in the Kappa test. Although the CIVand Kappa tests showed median agreement results, the need for a new round of evaluation was identified.

**Table 2 – Respondents according to leadership and management by team**

| **Variables** | **Control group** | | | | **Experimental Group** | | | |
| --- | --- | --- | --- | --- | --- | --- | --- | --- |
|  | **Pre-test** | | **Post-test** | | **Pre-test** | | **Post-test** | |
|  | n | % | n | % | n | % | n | % |
| **1. Faced with potential layoffs of nursing professionals due to the salary floor, how would you, as a leader, address this issue to keep the team motivated and engaged?** | | | | | | | | |
|  | n | % | n | % | n | % | n | % |
| Ignore the situation, focusing only on clinical aspects. | 0 | 0.0 | 0 | 0.0 | 00 | 0.0 | 0 | 0.0 |
| Seek open dialogue, exploring collaborative solutions. | 7 | 100.00 | 7 | 100.0 | 7 | 100.0 | 7 | 100.0 |
| Adopt an authoritarian stance to avoid salary discussions. | 0 | 0.0 | 0 | 0.0 | 00 | 0.0 | 0 | 0.0 |
| Delegate problem resolution to administration, without getting involved. | 0 | 0.0 | 0 | 0.0 | 00 | 0.0 | 0 | 0.0 |
| **2. Given signs of stress in the team, how would you promote an organizational culture that effectively addresses workload and preserves professional well-being?** | | | | | | | | |
|  | n | % | n | % | n | % | n | % |
| Implement a single stress management program for the entire team. | 0 | 0.0 | 0 | 0.0 | 00 | 0.0 | 0 | 0.00 |
| Ignore the stress, focusing only on clinical goals. | 0 | 0.0 | 0 | 0.0 | 00 | 0.0 | 0 | 0.0 |
| Increase the workload to accelerate the adaptation process. | 0 | 0.0 | 0 | 0.0 | 0 | 0.0 | 0 | 0.0 |
| Customize stress management strategies based on individual team members' needs. | 7 | 100.00 | 7 | 100.00 | 7 | 100.00 | 7 | 100.00 |
| **3. In an environment where conflicts may arise from difficult ethical decisions, how would you, as a leader, mediate a conflict between team members with opposing views on allocating scarce resources?** | | | | | | | | |
|  | n | % | n | % | n | % | n | % |
| Avoid taking sides, letting the team resolve the conflict alone. | 0 | 0.0 | 0 | 0.0 | 0 | 0.0 | 0 | 0.0 |
| Promote open debate and encourage the joint search for ethical solutions. | 6 | 85.71 | 6 | 85.71 | 7 | 100.00 | 7 | 100.00 |
| Make a unilateral decision to avoid prolonging the conflict. | 1 | 14.29 | 1 | 14.29 | 0 | 0.0 |  | 0.0 |
| Ignore the conflict, focusing only on clinical issues. | 0 | 0.0 | 0 | 0.0 | 0 | 0.0 | 0 | 0.0 |
| **4. As a leader, how would you provide constructive feedback to team members who made medication errors, while simultaneously promoting professional development?** | | | | | | | | |
|  | n | % | n | % | n | % | n | % |
| Ignore the errors to avoid internal conflicts. | 0 | 0.0 | 0 | 0.0 | 0 | 0.0 | 0 | 0.0 |
| Provide feedback in an individual meeting, highlighting areas for improvement and offering support for development. | 7 | 100.0 | 7 | 100.0 | 7 | 100.0 | 7 | 100.0 |
| Publicly hold the involved professionals accountable, aiming to prevent future errors. | 0 | 0.0 | 0 | 0.0 | 0 | 0.0 | 0 | 00 |
| Exclude the involved professionals, considering the errors unacceptable. | 0 | 0.0 | 0 | 0.0 | 0 | 0.0 | 0 | 00 |
| **5. How do you define leadership?** | | | | | | | | |
|  | n | % | n | % | n | % | n | % |
| The process of influencing people's behavior to achieve objectives in specific situations. | 6 | 85.71 | 7 | 100 | 7 | 100.00 | 7 | 100.00 |
| The process of transforming the behavior of an individual or an organization. | 0 | 0.00 | 0 | 0.00 | 0 | 0.00 | 0 | 0.00 |
| The legitimate right to exercise power within the organization to obtain workers' obedience. | 0 | 0.00 | 0 | 0.00 | 0 | 0.00 | 0 | 0.00 |
| I think it would be more of a mix between the first and second options. | 1 | 14.29 | 0 | 0.00 | 0 | 0.00 | 0 | 0.00 |
| **6. Do you consider yourself a leader?** | | | | | | | | |
|  | n | % | n | % | n | % | n | % |
| Yes | 0 | 0.00 | 3 | 42.86 | 3 | 42.86 | 4 | 57.14 |
| No | 7 | 100.0 | 4 | 57.14 | 4 | 57.14 | 3 | 42.86 |
| **7. Select the interpersonal skills you consider necessary for a leader?** | | | | | | | | |
|  | n | % | n | % | n | % | n | % |
| Communication skills. | 2 | 28.58 | 2 | 28.58 | 3 | 42.86 | 1 | 14.29 |
| Skill in giving and receiving feedback*.* | 0 | 0 | 0 | 0 | 0 | 0 | 0 | 0 |
| Skill in gaining power and exerting influence. | 0 | 0 | 0 | 0 | 0 | 0 | 0 | 0 |
| All the skills mentioned above. | 5 | 71.45 | 4 | 71.45 | 4 | 57.14 | 6 | 85.71 |

At a significance level of 5%, it was possible to observe that the results showed that there were no significant changes in the responses between the pre-test and the post-test, either for the control group or for the experimental group. For example, in the questions about the approach to situations involving stress in the team or about the way to provide feedback after medication errors, 100% of the participants in both groups maintained the same response at both evaluation times, resulting in p-values ​​of 1.000, indicating that there was no significant difference.

Other questions, such as the perception of leadership or the approach to ethical conflicts, also did not show significant changes in the responses, with p-values ​​that were above 0.05, such as 0.131 for the question on ethical conflicts in the experimental group and 0.343 for the perception of leadership in the control group. This suggests that, for these items, there was no change in the participants' attitudes or behaviors between the pre-test and the post-test.

**Table 4.** Comparison of item means for "Developing Skills for Leadership" [QUAPEEL items] in the pre- and post-test, using the Wilcoxon signed-rank test for paired samples for the experimental group.

| **Items** |  | | Pre-test | |  | | Post-test | |  |
| --- | --- | --- | --- | --- | --- | --- | --- | --- | --- |
| **Leadership practice** | Mean | *Median* | | *Std. Dev.* | Mean | *Median* | | *Std. Dev.* | *P* |
| 4.1. I know how to listen to those I lead. | 4.71 | 5.00 | | 0.49 | 4.57 | 5.00 | | 0.54 | 0.317 |
| 4.2. I manage to maintain the interest of those I lead in maintaining and continuing dialogue. | 3.43 | 4.00 | | 0.79 | 4.00 | 4.00 | | 0.89 | 0.102 |
| 4.3. I transmit guidance and advice to those I lead, addressing their professional needs. | 4.00 | 4.00 | | 1.00 | 4.17 | 4.00 | | 0.75 | 1.000 |
| 4.4. I use verbal communication and pay attention to non-verbal communication in dialogue with those I lead. | 4.29 | 5.00 | | 0.95 | 4.57 | 5.00 | | 0.54 | 0.317 |
| 4.5. I contribute to effective communication in work relationships with those I lead. | 4.00 | 4.00 | | 0.58 | 4.50 | 4.50 | | 0.55 | 0.083 |
| 4.6. I give guidance to those I lead and demonstrate how tasks should be performed, according to their needs. | 3.86 | 4.00 | | 0.90 | 4.29 | 4.00 | | 0.49 | 0.083 |
| 4.7. I clarify doubts of those I lead regarding their tasks. | 4.14 | 4.00 | | 0.90 | 4.71 | 5.00 | | 0.49 | 0.102 |
| 4.8. I recognize and value those I lead for what they do or how they behave. | 4.57 | 5.00 | | 0.79 | 4.43 | 4.00 | | 0.54 | 0.705 |
| 4.9. I redirect those I lead, showing a new path forward when they do not meet expected performance. | 4.00 | 4.00 | | 0.82 | 4.71 | 5.00 | | 0.49 | 0.102 |
| 4.10. I periodically monitor the performance of those I lead. | 4.25 | 4.50 | | 0.96 | 4.17 | 4.00 | | 0.75 | 0.655 |
| 4.11. I encourage the practice of feedback with those I lead. | 4.50 | 5.00 | | 0.84 | 4.33 | 4.00 | | 0.52 | 0.564 |
| 4.12. I exert influence on those I lead, expanding competencies in favor of effective results. | 4.00 | 4.00 | | 0.89 | 4.57 | 5.00 | | 0.54 | 0.102 |
| 4.13. I share decisions with those I lead. | 4.43 | 4.00 | | 0.54 | 4.83 | 5.00 | | 0.41 | 0.157 |
| 4.14. I delegate activities to those I lead, sharing responsibilities. | 4.17 | 4.00 | | 0.75 | 4.50 | 5.00 | | 0.84 | 0.257 |
| 4.15. I take responsibility for the development of those I lead. | 4.50 | 4.50 | | 0.55 | 4.50 | 4.50 | | 0.55 | 1.000 |
| 4.16. I make myself available to assist those I lead when they are facing professional difficulties. | 4.14 | 4.00 | | 0.90 | 4.83 | 5.00 | | 0.41 | 0.180 |
| 4.17. I ask for the opinion of those I lead to alter a procedure or propose an operational change. | 4.00 | 4.00 | | 0.82 | 4.33 | 4.00 | | 0.52 | 0.655 |
| 4.18. I assist in defining goals for each person I lead on my team. | 4.14 | 4.00 | | 0.69 | 4.67 | 5.00 | | 0.52 | 0.257 |
| 4.19. I periodically monitor the results presented by each person I lead. | 4.00 | 4.00 | | 0.89 | 4.40 | 5.00 | | 1.34 | 0.564 |
| 4.20. I agree on the necessary deadline with each person I lead for goals to be achieved. | 4.20 | 5.00 | | 1.09 | 4.17 | 4.50 | | 1.17 | 0.655 |

The study assessed leadership practices using 20 items, comparing the results obtained in the pre- and post-test of the post-test group of 14 participants. The items with the highest mean scores in the pre-test were "I know how to listen to my subordinates" (M = 4.71, SD = 0.49) and "I recognize and value my subordinates for what they do or how they behave" (M = 4.57, SD = 0.79), suggesting that active listening and recognition of subordinates were already well evaluated before the intervention. In the post-test, the item "I am available to help my subordinates when they are facing some professional difficulty" showed an increase in the mean, reaching 4.83 (SD = 0.41).

The analysis using the Wilcoxon test revealed that, despite the increase in the mean scores in several items, there were no statistically significant differences in most cases (p > 0.05). However, items such as "I contribute to effective communication in work relationships with subordinates" and "I provide subordinates with guidance and demonstrations of how tasks should be performed" presented values ​​of p = 0.083, close to the 10% significance level, suggesting trends for improvement that could be more evident in a larger sample.

**Table 5.** Comparison of item means for "Developing Skills for Leadership" [QUAPEEL items] in the pre- and post-test, using the Wilcoxon signed-rank test for paired samples for the control group.

| **Items** |  | | Pre-test | |  | | Post-test | |  |
| --- | --- | --- | --- | --- | --- | --- | --- | --- | --- |
| **Leadership practice** | Mean | *Median* | | *Std. Dev.* | Mean | *Median* | | *Std. Dev.* | *P* |
| 4.1. I know how to listen to those I lead. | 4.14 | 4.00 | | 0.69 | 4.71 | 5.00 | | 0.49 | 0.046 |
| 4.2. I manage to maintain the interest of those I lead in maintaining and continuing dialogue. | 3.71 | 4.00 | | 0.95 | 3.86 | 4.00 | | 0.38 | 0.564 |
| 4.3. I transmit guidance and advice to those I lead, addressing their professional needs. | 3.43 | 3.00 | | 0.98 | 4.00 | 4.00 | | 0.58 | 0.194 |
| 4.4. I use verbal communication and pay attention to non-verbal communication in dialogue with those I lead. | 4.29 | 4.00 | | 0.49 | 4.14 | 4.00 | | 0.69 | 0.564 |
| 4.5. I contribute to effective communication in work relationships with those I lead. | 4.00 | 4.00 | | 0.58 | 4.43 | 5.00 | | 0.79 | 0.083 |
| 4.6. I give guidance to those I lead and demonstrate how tasks should be performed, according to their needs. | 3.14 | 3.00 | | 0.69 | 4.43 | 4.00 | | 0.54 | 0.024 |
| 4.7. I clarify doubts of those I lead regarding their tasks. | 4.14 | 4.00 | | 0.90 | 4.43 | 4.00 | | 0.54 | 0.157 |
| 4.8. I recognize and value those I lead for what they do or how they behave. | 4.57 | 5.00 | | 0.54 | 4.57 | 5.00 | | 0.54 | 1.000 |
| 4.9. I redirect those I lead, showing a new path forward when they do not meet expected performance. | 3.71 | 4.00 | | 0.49 | 4.00 | 4.00 | | 0.58 | 0.317 |
| 4.10. I periodically monitor the performance of those I lead. | 3.86 | 4.00 | | 0.38 | 4.00 | 4.00 | | 0.82 | 0.564 |
| 4.11. I encourage the practice of feedback with those I lead. | 4.43 | 5.00 | | 0.79 | 4.29 | 4.00 | | 0.76 | 0.785 |
| 4.12. I exert influence on those I lead, expanding competencies in favor of effective results. | 4.14 | 4.00 | | 0.69 | 4.00 | 4.00 | | 0.82 | 0.705 |
| 4.13. I share decisions with those I lead. | 4.29 | 4.00 | | 0.49 | 4.14 | 4.00 | | 0.69 | 0.564 |
| 4.14. I delegate activities to those I lead, sharing responsibilities. | 4.14 | 4.00 | | 0.90 | 4.29 | 4.00 | | 0.49 | 0.705 |
| 4.15. I take responsibility for the development of those I lead. | 3.86 | 4.00 | | 0.69 | 4.14 | 4.00 | | 0.69 | 0.480 |
| 4.16. I make myself available to assist those I lead when they are facing professional difficulties. | 4.43 | 5.00 | | 0.79 | 4.83 | 5.00 | | 0.41 | 0.180 |
| 4.17. I ask for the opinion of those I lead to alter a procedure or propose an operational change. | 4.43 | 5.00 | | 0.79 | 4.43 | 4.00 | | 0.54 | 1.000 |
| 4.18. I assist in defining goals for each person I lead on my team. | 4.00 | 4.00 | | 0.58 | 4.14 | 4.00 | | 0.69 | 0.317 |
| 4.19. I periodically monitor the results presented by each person I lead. | 4.00 | 4.00 | | 1.00 | 4.00 | 4.00 | | 0.82 | 1.000 |
| 4.20. I agree on the necessary deadline with each person I lead for goals to be achieved. | 4.43 | 5.00 | | 0.79 | 3.83 | 4.00 | | 0.41 | 0.083 |

Among the items analyzed, it was observed that "I know how to listen to subordinates" showed an increase in the mean from 4.14 to 4.71, with a reduction in the standard deviation from 0.69 to 0.49, suggesting not only an improvement in the perception of this ability, but also greater uniformity in the responses. Likewise, "I give subordinates guidance and demonstrations of how tasks should be performed" had a significant increase in the mean, from 3.14 to 4.43, accompanied by a reduction in the standard deviation from 0.69 to 0.54, indicating a more homogeneous perception of this improvement. "I contribute to effective communication in work relationships with subordinates" had an increase in the mean from 4.00 to 4.43, with a slight increase in dispersion (standard deviation from 0.58 to 0.79), suggesting greater variation in perceptions after the intervention.

The item "I periodically monitor the performance of my subordinates" maintained practically the same average (3.86 to 4.00), but its standard deviation increased from 0.38 to 0.82, indicating a greater variation among participants in the post-test. On the other hand, the item "I agree on the time frame necessary for goals to be achieved" showed a decrease in the average, from 4.43 to 3.83, with a reduction in variability (standard deviation from 0.79 to 0.41), suggesting a change in the participants' perception of this practice.

Statistical analysis was performed using the Wilcoxon test for paired samples, adopted due to the lack of normality in the data. The results indicate that, despite some improvements in the mean scores, only two items presented statistically significant differences (p < 0.05): "I know how to listen to subordinates" (p = 0.046), evidencing a significant improvement in listening ability, and "I give subordinates guidance and demonstrations of how tasks should be performed" (p = 0.024), indicating advances in practical guidance to subordinates. Other items presented p-values ​​greater than 0.05, suggesting a lack of statistical significance, although some approached the adopted level, such as "I contribute to effective communication in work relationships" (p = 0.083) and "I agree on the necessary time frame for goals to be achieved" (p = 0.083). The lack of statistical significance for most items can be attributed to the small sample size (n = 14), limiting the statistical power of the test. However, the results suggest trends of improvement that could become statistically significant in a study with a larger sample.
