# Supplementary material for: Effect of an Online Module on Leadership on the Knowledge Acquisition of Nursing Students: A Pilot Randomized Clinical Trial Study
Source: J Nurs Manag. 2025 Sep 9;2025:3769545. doi: 10.1155/jonm/3769545 (PMC12440644; doi:10.1155/jonm/3769545)

Analysed (n=07)

Lost to follow-up (n=0)

Discontinued intervention (n=0)

Allocated to the Experimental Group (n=07)

Received allocated intervention (n=07)

Did not receive allocated intervention (give reasons) (n=0)

Allocated to the Control Group (n=07)

Received allocated intervention (n=07)

Did not receive allocated intervention (give reasons) (n=0)

Lost to follow-up (n=0)

Discontinued intervention (n=0)

Analysed (n=07)

Randomized (n=14)

Assessed for eligibility (n=36)

Excluded (n=22)

Not meeting inclusion criteria (n=0)

Declined to participate (n=22)

Other reasons (n=0)

**Enrollment**

**Allocation**

**Follow-Up**

**Analysis**

**CONSORT 2010 Flow Diagram**


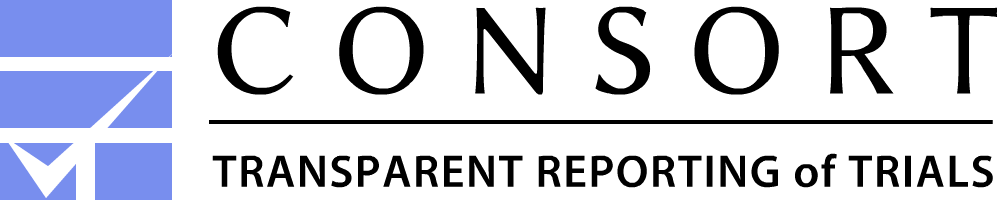

Supplement: Supporting Information 5 — S5: CONSORT Flow diagram. [file 3769545.f5.docx]
