# Supplementary material for: Effect of an Online Module on Leadership on the Knowledge Acquisition of Nursing Students: A Pilot Randomized Clinical Trial Study
Source: J Nurs Manag. 2025 Sep 9;2025:3769545. doi: 10.1155/jonm/3769545 (PMC12440644; doi:10.1155/jonm/3769545)
Supplement: Supporting Information 6 — S6: Certificate English Editing. [file 3769545.f6.pdf]

NEW HORIZONS
ACADEMIC TRANSLATIONS

CERTIFICATE OF ENGLISH LANGUAGE EDITING

This certificate confirms that the manuscript listed below has been professionally proofread and edited for grammar, spelling, punctuation, clarity, and overall readability by New Horizons Academic Translations. The editing was carried out by an English speaker with expertise in academic writing, ensuring that the language meets the standards required for scholarly publication. Our company details* are provided at the end of this document for further information, if needed.

Manuscript title: “Effect of an online module on leadership on the knowledge acquisition of nursing students: a pilot randomized clinical trial study”

Date: August 7, 2025

Certificate Nº.: jf25v2

Vanessa Emille C. S. Freire
Responsible translator and owner

*New Horizons is a company specialized in the translation, review, and preparation of manuscripts in Portuguese, English and Spanish. Our company offers editing, translation, formatting, and illustration services for academic purposes. All translations are performed by one or more professional scientific translators/editors with PhD and proficiency in Portuguese, English and Spanish.

New Horizons
CNPJ: 37.734.574/0001-01
Rua Joao Cordeiro, 3069, Joaquim Tavora, 60110-535, Fortaleza, CE, Brazil
Phone: 55 85 99667-8240
E-mail: contato@newhorizons.net.br
